# The dual role of leukotrienes in the tumor microenvironment: balancing pro-tumorigenic and anti-tumor immunity

**DOI:** 10.1080/15384047.2026.2692168

**Published:** 2026-06-23

**Authors:** Andrew Thake, Maria Caruso, Sebastian Fernandez-Bussy, Keith Sacco

**Affiliations:** a University of Edinburgh, Edinburgh, UK; b Mater Dei Hospital, Msida, Malta; c Division of Pulmonary, Allergy and Sleep Medicine, Mayo Clinic, Jacksonville, FL, United States; d The Immunology Clinic, Msida, Malta

**Keywords:** Leukotrienes, precision immunotherapy, cancer immunology, tumor microenvironment, biomarkers, 5-lipoxygenase, leukotriene receptor antagonists, targeted therapy

## Abstract

**Background:**

Leukotrienes are bioactive lipid mediators produced via the 5-lipoxygenase (5-LO) pathway and are essential for inflammatory signaling in the tumor microenvironment (TME), playing roles in angiogenesis, immune modulation and metastatic progression.

**Objective:**

This review evaluates the role of leukotriene signaling in cancer progression and highlights biomarker-guided therapeutic strategies targeting these pathways in the TME.

**Methods:**

A narrative review of preclinical, clinical, and epidemiological studies was performed, with prespecified inclusion criteria and prioritization of recent evidence.

**Results:**

Leukotriene-mediated signaling correlates with biomarkers such as CysLT1 receptor expression, *β*-catenin activation, and HIF-1α signaling. In preclinical models, pharmacological agents including montelukast, zileuton, and FLAP antagonists suppress tumor growth, and restore apoptotic signaling; current clinical evidence remains largely observational and pharmacoepidemiologic.

**Conclusion:**

Targeting this pathway represents a promising avenue in oncology, with potential to enhance precision medicine through biomarker-guided patient stratification. Further clinical validation is warranted to translate these findings into therapeutic benefit.

## Introduction

Leukotrienes are bioactive lipid mediators produced through the lipoxygenase (LOX) pathway and play an important role in arachidonic acid metabolism. They are historically known for their role in acute inflammation and allergy; however, leukotrienes at elevated concentrations are increasingly understood to facilitate chronic, subclinical inflammatory responses. Recent studies further suggest that leukotrienes function as regulators of neoplastic disease progression and modulators of immune responses.^1^

Leukotrienes occupy a biologically distinct niche among the eicosanoids. Arachidonic acid is metabolized along two principal enzymatic branches: the cyclooxygenase (COX-1/COX-2) branch, which generates prostaglandins (e.g., PGE₂) and thromboxanes signaling through the EP, DP, IP, FP, and TP G-protein–coupled receptors, and the 5-lipoxygenase (5-LO)/FLAP branch, which generates the leukotrienes.^2^ ^,^ ^3^ Within the latter branch, leukotrienes are functionally divided from the pro-resolving lipoxins, which are also 5-LO–derived yet anti-inflammatory.^4^ ^,^ ^5^ Three features make leukotriene signaling distinctive within the TME and motivate this review: it is receptor-restricted (acting through the dedicated BLT and CysLT receptor families); the cysteinyl leukotriene LTE₄ is comparatively stable and therefore persists longer within tissues; and the output is functionally bidirectional, capable of either promoting tumor growth or supporting antitumor immunity depending on the species, receptor, and cellular context.^6^ This biological significance—a single pathway that can drive opposing outcomes—distinguishes leukotrienes from the broadly pro-tumorigenic COX/PGE₂ axis and underlies the precision-targeting rationale developed below.^5^ ^,^ ^7^ ^,^ ^8^

In the neoplastic milieu, this low-grade inflammation is predominantly localized within the tumor microenvironment.^1^ ^,^ ^2^ Leukotrienes adeptly modify the microenvironment in ways that may either facilitate or impede tumor progression.^9^ This biological conundrum highlights the necessity of comprehending leukotriene signaling within the TME to formulate precision immunotherapies. In the tumor microenvironment, leukotrienes act as paracrine messengers that facilitate communication between immune cells and malignant cells.^2^ The biosynthesis of leukotrienes splits into two main functional categories: the cysteinyl leukotrienes (CysLTs), encompassing LTC₄, LTD₄, and LTE₄, along with chemotactic leukotriene B₄ (LTB₄). CysLTs generally promote tumor growth and immune suppression while, conversely, LTB₄ facilitates the recruitment of anti-tumor immune cells such as CD8⁺ T lymphocytes.^2^ These mediators are primarily produced by immune cells such as macrophages, neutrophils, mast cells, and eosinophils. Their synthesis depends on the activation and subcellular localization of 5-lipoxygenase (5-LO), which catalyzes the conversion of arachidonic acid into leukotriene intermediates.^3^ ^,^ ^4^

This review provides a comprehensive overview of leukotriene biosynthesis, receptor signaling, and paradoxical effects in cancer. Importantly, we integrate current mechanistic and clinical evidence to propose a biomarker-guided therapeutic framework that distinguishes tumor-promoting from immune-protective leukotriene signaling. Understanding leukotriene signaling in the TME is critical for developing precision immunotherapy and anti-cancer strategies. We argue that therapeutic targeting of leukotriene pathways requires receptor-specific modulation to maximize anti-tumor immunity while limiting tumor-promoting effects.

In contrast to previous reviews that have cataloged the involvement of leukotrienes in cancer, the distinguishing contribution of the present work is an explicit, receptor-resolved and biomarker-guided framework (consolidated in Table 1 and summarized schematically in Figure 2). Rather than treating “leukotriene inhibition” as a single strategy, we operationally separate tumor-promoting cysteinyl-leukotriene signaling from immune-protective LTB₄–BLT1 signaling and translate this separation into stratification-ready targeting hypotheses. Throughout, we explicitly label evidence as preclinical (in vitro or animal) or as clinical/epidemiologic, so that these findings should not be interpreted as evidence of established clinical efficacy.

**Table 1.** Biomarker-guided therapeutic matrix.

Target/Signaling Pathway	Malignancy	Therapeutic Context	Potential Agents	Mechanistic Outcome	References
CysLT1R	CML, Prostate, Breast, Lung, Pancreatic	High proliferation/viability	Montelukast, other CysLT1 antagonists	↓ Proliferation, ↑ apoptosis	[10–16]
CysLT2R	Colon, Uveal melanoma	Differentiation stage/CYSLTR2 mutation	Agonizts or modulator approaches	Promote differentiation, counteract oncogenic signaling	[17–20]
LTD4 signaling	Monocytic leukemia, Colon	Early signaling/apoptosis resistance	LTD4 antagonists	Restore apoptosis, ↓ MAPK activation	[21–25]
5-LO/Leukotriene synthesis	CML, Prostate	High proliferation	MK886, Montelukast	Induce apoptosis, ↓ proliferation	[12,26]
*β*-catenin/NFκB	Colon	Wnt-active tumors	CysLT1R antagonists, leukotriene pathway inhibitors	↓ proliferation & migration, modulate mitochondrial activity	[22,24]
HIF-1α/Hypoxia	Prostate	Hypoxic microenvironment	CysLT1R antagonists	↓ HIF-1α expression, ↑ sensitivity to therapy	[14]
MMP 2/9	Glioblastoma	Invasive stage	Leukotriene antagonists	↓ invasion & metastasis potential	[27]
mMICA/immune ligands	Hepatoma	Immune evasion	Montelukast/Pranlukast	↑ immune recognition	[28]
Angiogenic markers (Calpain 2)	Colorectal	Angiogenesis-prone tumors	Quininib	↓ angiogenesis	[29]
CYSLTR2 mutation (L129Q)	Uveal melanoma	Mutation-driven tumors	CYSLTR2 modulators ± CysLT1R antagonists	Stabilize disease/↓ tumor growth	[17,19,20]

Abbreviations: CysLT = cysteinyl leukotriene; CysLT1R = cysteinyl leukotriene receptor 1; CysLT2R = cysteinyl leukotriene receptor 2; LTD₄ = leukotriene D₄; 5-LO = 5-lipoxygenase; FLAP = 5-lipoxygenase-activating protein; NFκB = nuclear factor kappa-light-chain-enhancer of activated B cells; *β*-catenin = beta-catenin (Wnt pathway transcription co-activator); HIF-1α = hypoxia-inducible factor 1-alpha; MMP = matrix metalloproteinase; mMICA = membrane-bound MHC class I chain-related protein A; CYSLTR2 = cysteinyl leukotriene receptor 2 (gene/protein); L129Q = leucine-to-glutamine substitution at position 129; CML = chronic myeloid leukemia; ICI = immune checkpoint inhibitor; ALOX5 = arachidonate 5-lipoxygenase gene; PGE₂ = prostaglandin E₂; EMT = epithelial-to-mesenchymal transition; MK886 = FLAP inhibitor; Quininib = small-molecule angiogenesis inhibitor (calpain-2/TIE-2); ↑ = increased; ↓ = decreased.

## Materials and methods

A narrative review methodology was applied. Peer-reviewed studies, experimental models, clinical trials, and epidemiological research examining leukotriene biosynthesis, receptor signaling, and therapeutic modulation in cancer were included. Emphasis was placed on studies linking biomarkers of leukotriene activity (e.g., CysLT1, BLT1, *β*-catenin, HIF-1α, CYSLTR2 mutations) to potential therapeutic interventions. Search terms included ‘leukotrienes’, ‘tumor microenvironment’, ‘5-lipoxygenase’, and ‘cancer immunology’. Literature searches were performed using PubMed, Scopus, and Web of Science databases, focusing on publications from 2000–2025.

To improve transparency and reproducibility, we applied the following criteria. Inclusion: English-language, peer-reviewed primary studies (in vitro, in vivo, or human), systematic reviews, and authoritative regulatory communications addressing 5-LO/leukotriene biosynthesis, leukotriene receptor signaling, or pharmacological modulation in the context of malignancy or the tumor microenvironment. Exclusion: non-peer-reviewed material, studies without an oncologic or tumor-immunologic readout, and reports for which a mechanistic or clinical link to leukotriene signaling could not be established. Records were screened by title and abstract and then by full text; reference lists of key articles were hand-searched for additional sources. Where evidence was abundant, we prioritized recent (2015–2026) and higher-quality studies (clinical and large preclinical datasets) while retaining seminal earlier mechanistic work to preserve historical context. As this is a narrative rather than systematic review, no formal risk-of-bias scoring or meta-analysis was performed.

## Leukotriene biology in the TME

Leukotriene biosynthesis begins with the release of arachidonic acid from membrane phospholipids. This substrate is subsequently metabolized by the enzyme 5-lipoxygenase (5-LO) in association with the 5-lipoxygenase-activating protein (FLAP), producing the unstable intermediate leukotriene A₄ (LTA₄), which acts as a precursor for several leukotriene species.^1^ As a central precursor, LTA₄ sits at a metabolic crossroads and can be funneled into two primary enzymatic pathways. In one direction, LTA₄ hydrolase converts the intermediate into leukotriene B₄ (LTB₄), a key chemotactic mediator responsible for driving immune cell recruitment.

Alternatively, LTC₄ synthase directs the formation of leukotriene C₄ (LTC₄), which serves as the parent molecule for the subsequent production of LTD₄ and LTE₄. Within this group of cysteinyl leukotrienes, LTE₄ stands out as the most chemically stable metabolite. Because of this stability, LTE₄ can persist longer within the tumor microenvironment.^1–3^ The biological impact of 5-LO rests on its location within the cell. Since the enzyme is highly dynamic and translocates between distinct subcellular compartments, its spatial sensitivity effectively dictates the final metabolic output. For example, translocation towards the nucleus or Golgi apparatus favors the production of LTB₄. However, when 5-LO remains seated in the cytosol, the pathway tends to produce CysLTs or lipoxin A₄, the latter of which exhibits anti-inflammatory properties.^4^

**Figure 1.**
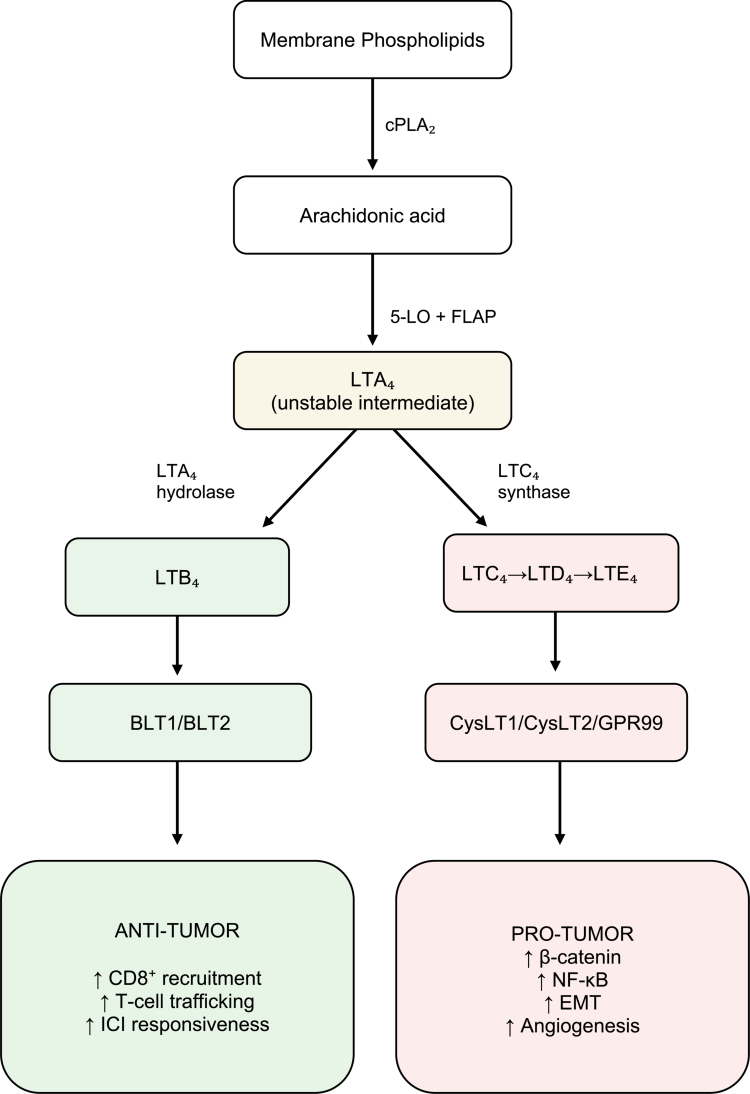
Leukotriene biosynthesis and receptor landscape within the tumor microenvironment. Arachidonic acid is converted by 5-lipoxygenase (5-LO) and 5-lipoxygenase-activating protein (FLAP) into leukotriene A₄ (LTA₄), which is subsequently metabolized into leukotriene B₄ (LTB₄) or cysteinyl leukotrienes (LTC₄, LTD₄, and LTE₄). LTB₄–BLT signaling promotes antitumor immunity through recruitment of cytotoxic T cells and enhanced responsiveness to immune checkpoint inhibition. In contrast, cysteinyl leukotriene signaling promotes oncogenic signaling, epithelial–mesenchymal transition, angiogenesis, and tumor progression. The context-dependent duality of leukotriene signaling provides a rationale for receptor-selective and biomarker-guided therapeutic targeting.

Mechanistically, 5-LO activity is tightly regulated rather than constitutive. Cellular activation raises intracellular calcium and triggers translocation of 5-LO to the nuclear envelope, where FLAP presents arachidonic acid to the enzyme; this membrane docking is a prerequisite for efficient leukotriene synthesis and is the molecular target of FLAP antagonists. 5-LO is further modulated by phosphorylation—ERK1/2 and MK2 (MAPKAPK2) phosphorylation generally promotes activity, whereas protein kinase A–mediated phosphorylation is inhibitory—and by redox state and the nuclear-versus-cytosolic localization noted above. In cancer, sustained ALOX5 (5-LO) expression and FLAP availability therefore convert episodic inflammatory signaling into chronic leukotriene flux, linking the enzyme directly to proliferative, angiogenic, and immunomodulatory programs in the TME. The overall pathway and its divergent receptor outputs are summarized in Figure 1.

Even at nanomolar concentrations, the pathway exhibits significant metabolic cooperation through transcellular synthesis. While certain stromal cells within the tumor microenvironment may lack the endogenous 5-LO machinery, they can still intercept LTA₄ secreted by neighboring cells. This intercellular exchange allows the TME to function as a collective biosynthetic unit, broadening the inflammatory profile beyond single-cell-type capabilities.^5^ ^,^ ^6^

To understand the paradox mentioned in the introduction, one must examine the receptor landscape. Leukotrienes mediate their effects through two classes of G-protein–coupled receptors (GPCRs). Leukotriene B₄ (LTB₄) binds to BLT1 (high affinity) and BLT2 (low affinity) receptors. BLT1 signaling is associated with anti-tumor CD8⁺ T-cell recruitment, whereas BLT2 signaling has been implicated in oncogenesis. Conversely, cysteinyl leukotrienes (CysLTs) signal through CysLT1, CysLT2, and CysLTe (GPR99).^5^ ^,^ ^8^ ^,^ ^30^

In addition to their receptor-mediated effects, lipoxygenases (LOs) and their increased leukotriene flux exert diverse and context-specific influences in multiple pathological states such as cancer, type 2 diabetes, asthma, and Alzheimer’s disease.^7^

## Pro-tumorigenic effects of leukotrienes

Elevated expression of 5-lipoxygenase (5-LO) and increased leukotriene signaling have been observed across multiple cancer types.^31^ ^,^ ^32^ Leukotriene mediators drive oncogenesis by modulating critical cellular processes, including apoptosis, migration, and invasion. Furthermore, 5-LO and leukotriene signaling actively shape the tumor microenvironment by inducing immune cell recruitment and activation, alongside the production of various growth factors, angiogenic agents, and proinflammatory mediators.^33^

The LTD₄/CysLT1 signaling pathway contributes significantly to oncogenic progression, with *β*-catenin serving as a central regulatory component. Upon binding of LTD₄ to the CysLT1 receptor, a cascade is initiated that induces the nuclear translocation of *β*-catenin, particularly evidenced in intestinal and colon cancer cell lines such as Int 407 and HCT116.^21^ Once localized within the nucleus, *β*-catenin activates TCF/LEF transcription factors to upregulate target genes that drive enhanced cellular proliferation and migration, including cyclin D1 and c-Myc. This mitogenic effect can be specifically attenuated through pretreatment with CysLT1 receptor antagonists, such as ZM198,615.^34^

CysLT1-driven *β*-catenin signaling intersects with canonical Wnt signaling pathways; *β*-catenin accumulation activates NF-κB (p65) with downstream ROS production; LTD₄ stimulates PI3K/AKT and ERK/MAPK survival signaling; and the 5-LO branch is metabolically coupled to the COX-2/PGE₂ pathway, such that perturbation of one arm may alter activity within the other. These interconnected signaling networks contribute to tumor progression and may influence therapeutic response and resistance, as discussed below. The CysLT pathway may also promote resistance to apoptosis through Bcl-2 upregulation, potentially involving mitochondrial localization of free *β*-catenin.^7^ ^,^ ^32^ ^,^ ^35^

Moreover, non-metastatic breast cancer cells produce 5-LO metabolites, particularly LTB₄, which can activate tumor-evoked regulatory B cells (tBregs) through PPARα, promoting immune evasion and metastasis.^7^ This effect is largely abolished when tBregs are pretreated ex vivo with the 5-LO inhibitor MK886, highlighting that 5-LO activity is critical not only in cancer cells but also within B cells. Further pharmacological studies demonstrate that 5-LO/LT/PPARα signaling in tBregs is needed for the generation of pro-metastatic FOXP3⁺ regulatory T cells and for suppressing cytotoxic CD8⁺ T cells, illustrating how leukotriene pathways simultaneously support tumor progression and modulate anti-tumor immunity.^34^ ^,^ ^35^ PPARα is also modulated by LTE₄, a stable cysteinyl leukotriene, which contributes to tumor-promoting effects within the microenvironment.

Immune stromal cells in esophageal adenocarcinoma show higher 5-LO and other leukotriene-producing enzymes than normal tissue, suggesting that 5-LO–derived leukotrienes contribute to the tumor microenvironment during progression.^36^ Similarly, overexpression of 12-LO and 5-LO has been reported in lung, renal, breast, prostate, colorectal, and pancreatic tumors compared to normal tissues.^7^

Leukotrienes further promote tumorigenesis by facilitating epithelial-to-mesenchymal transition (EMT). This shift is important because EMT enables cancer cells to acquire invasive and metastatic properties. EMT is regulated by transcription factors such as ZEB, TWIST, and SNAIL, which drive progression from primary tumor growth to invasion, metastasis, colonization, and therapy resistance.^37^ At the level of invasion, cysteinyl-leukotriene-receptor antagonists reduce migration and invasion of glioblastoma cells with concomitant suppression of matrix metalloproteinases MMP-2 and MMP-9; notably, CysLT1 expression in A172 glioblastoma cells is induced 3.13-fold by interleukin-1β, and the median toxic concentration of these antagonists in glioma lies in the micromolar range (≈ 7–26 µM), a point relevant to drug repurposing discussed below.^27^ ^,^ ^37^ ^,^ ^38^ (preclinical).

5-LO signaling also mediates TME crosstalk via mast-cell–derived LTB₄, which recruits tumor-promoting neutrophils that enhance metastatic transformation and colonization of breast cancer cells through ERK activation. In murine models of polyposis, leukotriene-mediated ERK activation drives tumor progression, whereas pharmacological inhibition with zileuton reduces polyp burden and systemic inflammation. Genetic deletion or pharmacological ablation of 5-LO or LTA4H reduces tumor burden in K-ras–driven pancreatic ductal adenocarcinoma and human pancreatic xenografts, partly by decreasing TNF-*α* secretion. TNF-*α* acts in a context-dependent manner and, together with LTB₄, promotes tumor cell growth and metastasis.^7^ ^,^ ^36^ ^,^ ^37^

Leukotrienes also promote tumor angiogenesis and lymphatic vessel remodeling. Mechanistically, inhibition of 5-LO or LTB₄/BLT2 signaling blocks VEGF-A-driven angiogenesis (i.e., BLT2 acts upstream of VEGF-A in endothelial cells). LTC₄ and LTD₄, via CysLT2, increase blood vessel growth and permeability, supporting metastasis. LTB₄ stimulates lymphatic endothelial cell growth at low doses but can inhibit it at higher concentrations. This regulation of lymphatic vessels may also influence the effectiveness of immunotherapy.^7^ ^,^ ^39^

## Antitumor immunity—leukotrienes

Leukotriene signaling through the 5-LO pathway demonstrates both pro-tumorigenic and antitumor functions. Cysteinyl leukotrienes and LTB₄ act as strong chemoattractants for T cells within the tumor microenvironment.^7^ ^,^ ^9^ Experimental studies in murine cervical cancer models showed that BLT1 expression is critical for CD8⁺ T-cell recruitment, which mediates antitumor immunity. However, lung cancer cells implanted into 5-LO knockout mice developed into larger tumors compared with those in wild-type mice.

Together, these findings indicate that BLT1-driven CD8⁺ T-cell recruitment contributes to the antitumor effects of 5-LO signaling. Tumors in 5-LO knockout mice showed reduced infiltration of CD8⁺ T cells, NK cells, and CD4⁺ T cells. CD8⁺ T-cell neutralization in wild-type mice resulted in tumor sizes comparable to those observed in 5-LO-deficient mice, supporting a protective role for CD8⁺ T cells in tumor suppression.^40^

Within tumors, macrophage-derived leukotrienes are essential for attracting T cells. LTB₄, produced through the coordinated actions of 5-lipoxygenase and LTA4 hydrolase, acts as a potent chemoattractant for CD8⁺ T cells. This process works alongside CXCR3 ligands such as CXCL9 and CXCL10, which also guide T cells toward the tumor. Deleting either BLT1 or CXCR3 equally reduces T-cell infiltration, and dual knockouts show no additive effect, demonstrating that both pathways are required for efficient immune recruitment.^41^

The significance of this pathway is further highlighted by the role of LTB₄–BLT1 signaling in facilitating T-cell migration. Effective responses to PD-1 immunotherapy correlate with high levels of tumor-infiltrating lymphocytes. In melanoma, PD-1 blockade failed in BLT1⁻/⁻ mice due to a lack of T-cell recruitment; the absence of LTB₄-driven recruitment left no target cells for PD-1 antibodies to activate, resulting in complete treatment failure. These findings emphasize LTB₄ not only as a natural antitumor mechanism but also as a critical requirement for the clinical success of immune checkpoint therapies.^7^ ^,^ ^17^ ^,^ ^42^

The role of LTB₄ in T-cell recruitment is further supported by pharmacological studies. Inhibition of 5-LO with zileuton reduced polyp burden and systemic inflammation in murine models. Blocking 5-LO or LTA4H, either pharmacologically or genetically, reduced tumor growth in K-ras–driven pancreatic cancer and human xenograft models, partly by lowering TNF-*α* levels. Clinical trials using LTB₄ antagonists in lung cancer unexpectedly worsened disease progression, likely due to impaired T-cell infiltration rather than direct effects on tumor cells.^7^ ^,^ ^17^ ^,^ ^39^ ^,^ ^43^

## The 5-LO paradox

A major challenge in targeting leukotriene pathways therapeutically is the 5-LO paradox, whereby leukotriene signaling can both promote tumor growth and enhance anti-tumor immunity depending on cellular context. In the pathogenesis of leukemia, LTB₄ and LTC₄ act as mediators of pro-tumor activity. Conversely, a non-canonical, catalytically inactive form of 5-LO contributes to leukemic stem-cell maintenance through its interaction with *β*-catenin, thereby inhibiting Wnt signaling. Oncogenic fusion proteins including RUNX1-ETO9a, MLL-AF9, and AF4-MLL, alongside specific chromosomal abnormalities, elevate 5-LO expression.

5-LO also influences leukemic cells in the bone marrow. In BCR-ABL–driven CML mouse models, the absence of 5-LO prevents CML development through impaired leukemic stem-cell differentiation, proliferation, and long-term survival; pharmacological inhibition with zileuton produced comparable effects. In PML/RARα-positive acute myeloid leukemia and murine cancer stem-cell models, inhibition of PML/RARα-mediated Wnt activation was observed. Genetic knockout of 5-LO did not reproduce the inhibitory effects seen with pharmacological inhibitors; suppression of Wnt signaling was instead mediated by an enzymatically inactive form of 5-LO that binds *β*-catenin, preventing its nuclear translocation—indicating that non-canonical functions of 5-LO contribute to leukemogenesis.^44^ ^,^ ^45^

5-LO products such as LTB₄ also play a role in chemotaxis and inflammasome activation. In solid tumors, 5-LO can make the environment hostile to cancer growth; however, deleting 5-LO or blocking BLT1 impairs antitumor immunity. In melanoma, colon, and lung models, BLT1 knockout mice showed increased tumor size and fewer CD8⁺ T cells, and supplementing wild-type CD8⁺ T cells reversed these effects and improved checkpoint-inhibitor responses. BLT1 knockout germ-free mice did not develop tumors, showing that tumor control depends on LTB₄, MyD88 signaling, and the resultant microbiota. Similarly, BLT1 inhibitors in lung cancer aggravated tumor growth. These results reveal the paradox: 5-LO products can simultaneously oppose and support tumor progression.^5^ ^,^ ^41^

## Clinical and epidemiologic evidence

Human tumor biopsies and population-based studies have provided important clinical evidence for the role of leukotriene signaling in lung cancer. Examination of lung tumor biopsies revealed overexpression of 5-lipoxygenase and leukotriene A4 hydrolase compared with healthy lung tissue. (clinical, tissue-based) High 5-LOX levels correlate with advanced tumor stage and metastasis, indicating a potential role for leukotriene signaling in tumor-promoting inflammation. Recent studies indicate that leukotriene production in tumors primarily originates from myeloid cells such as neutrophils and macrophages, in which ALOX5 is often upregulated, contributing to an immunosuppressive microenvironment. The resultant myeloid-derived leukotrienes signal via CysLT₁ and BLT₁ receptors, supporting tumor proliferation and immune escape.^10^

A potential resolution to the 5-LOX paradox is explored in epidemiologic studies of lung carcinogenesis. (clinical/epidemiologic) A nationwide retrospective cohort study in Taiwan examined asthma patients treated with cysteinyl leukotriene receptor antagonists; in a cohort of 4,185 LTRA users compared with more than 20,000 non-users, researchers identified a dose-dependent decrease in general cancer risk, specifically lung cancer.^11^ Data from asthmatic U.S. veterans support these conclusions: regardless of age or smoking history, use of montelukast, zafirlukast, and zileuton was associated with a 17–22% decrease in the incidence of lung cancer.^46^ Leukotriene pathway inhibitors are an important pharmacological option because they can lower pro-tumor inflammation without impairing the host’s CD8⁺ T-cell immune response, supporting their potential as adjuvant or chemopreventive agents in high-risk populations.

Biomarker-level clinical correlates further support patient selection. In uveal melanoma, high CysLT1R expression correlates with poorer patient survival, and cognate antagonists modulate tumor-cell growth, secretome, and metabolism. (clinical-correlative and preclinical) Recurrent activating CYSLTR2 mutations (notably L129Q) occur in a minority of uveal melanomas and define a mutation-driven subset; a recent translational case report documented disease control with montelukast in a CYSLTR2-mutant metastatic uveal melanoma, providing early human proof-of-concept for receptor-resolved targeting. In prostate cancer models, montelukast suppresses HIF-1α translation under hypoxia, linking the CysLT1 biomarker to a tractable hypoxia-response node.^11–14^ (preclinical) These correlates are consolidated by malignancy in Table 1.

## Therapeutic outlook

Pharmacological intervention in the leukotriene pathway involves several strategies. FLAP antagonists prevent 5-LO activation, while agents such as zileuton act as 5-LO inhibitors that reduce leukotriene synthesis. Leukotriene receptor antagonists, such as montelukast and LY293111, prevent receptor-mediated signaling. In experimental models, these drugs slowed the growth of colon, lung, pancreatic, and prostate cancers. A number of natural substances affecting COX-2 and 5-LO signaling, including resveratrol, ginsenosides, s-allylmercaptocysteine, and curcumin, merit further consideration. By targeting leukotriene signaling pathways that promote tumor growth, existing pharmaceuticals may be repurposed for cancer treatment. Inhibition of 5-LO can reduce LTB₄-driven inflammation and angiogenesis within the tumor microenvironment while potentially preserving beneficial BLT1-mediated T-cell recruitment.^11,43–45^

A critical appraisal of the principal agents is warranted since efficacy, safety, and clinical-trial maturity differ substantially (Table 1). Montelukast is an oral, once-daily CysLT1 antagonist with extensive real-world tolerability data in asthma; however, in March 2020 the U.S. FDA added a boxed warning for serious neuropsychiatric events, including agitation, depression, and suicidality,^47^ a consideration that is non-trivial for any chronic oncologic dosing and for patient counseling. Critically, its anticancer evidence is preclinical and pharmacoepidemiologic; no completed phase III randomized controlled trial has established an oncologic benefit. Zileuton, a direct 5-LO inhibitor, carries a risk of hepatotoxicity that mandates serial monitoring of hepatic transaminases, and its short half-life and frequent dosing burden adherence. Zafirlukast is likewise associated with idiosyncratic hepatotoxicity. FLAP antagonists (e.g., the preclinical tool compound MK-886, and clinical FLAP inhibitors such as those evaluated in asthma) have not advanced into oncology trials and remain largely experimental in the cancer setting. In aggregate, the translational maturity of leukotriene-pathway agents in oncology is early, and enthusiasm should be tempered by the absence of randomized efficacy data.

The case for drug repurposing is nonetheless attractive: leukotriene modifiers are approved, inexpensive, orally bioavailable, and have well-characterized pharmacokinetics and safety, which lowers the barrier to early-phase oncology trials. A central caveat, however, is exposure: many anticancer effects in vitro require micromolar concentrations (e.g., the micromolar median toxic concentrations observed in glioma), whereas standard anti-asthma dosing achieves nanomolar receptor antagonism. Whether tumor-relevant exposures can be reached safely—potentially via dose optimization, local delivery, or nanoparticle/carrier-based formulations—is a key determinant of translational feasibility and an active area of formulation research.^33,38,48–50^

Several limitations and resistance mechanisms constrain clinical translation. First, eicosanoid class-switching: blockade of the 5-LO arm can shunt arachidonic acid toward COX-2/PGE₂, an independently pro-tumorigenic axis, potentially blunting benefit and arguing for dual or combination strategies. Second, receptor redundancy across BLT1/BLT2 and CysLT1/CysLT2/GPR99 permits compensatory signaling when a single receptor is blocked. Third, tumor and microenvironmental heterogeneity means the same intervention may be protective in one compartment and harmful in another. Fourth—and most important for safety—non-selective inhibition risks disrupting protective LTB₄–BLT1 immunity; this is not theoretical, as LTB₄-antagonist trials in lung cancer worsened outcomes. Off-target effects and the neuropsychiatric profile of montelukast add further constraints. These considerations reinforce a receptor-selective rather than pathway-wide approach.

To address the complexity of leukotriene signaling in cancer, we propose a biomarker-guided therapeutic matrix (Table 1) that aligns specific leukotriene pathways with targeted interventions, enabling a precision-based approach that selectively inhibits tumor-promoting mechanisms while preserving anti-tumor immune function. Table 1 lists methods for specifically blocking oncogenic leukotriene signaling—*β*-catenin/NF-κB inhibition in Wnt-active tumors, CysLT1R antagonists in highly proliferative tumors, and CYSLTR2 modulators in mutation-driven cancers—while antitumor immune activity is maintained. Eicosanoid compensation, tumor heterogeneity, and possible off-target effects limit clinical translation despite preclinical promise; for instance, pro-tumor PGE₂ levels may unintentionally rise due to LTB₄ inhibition. Thus, combination therapy and optimal patient selection are essential.^11^

**Figure 2.**
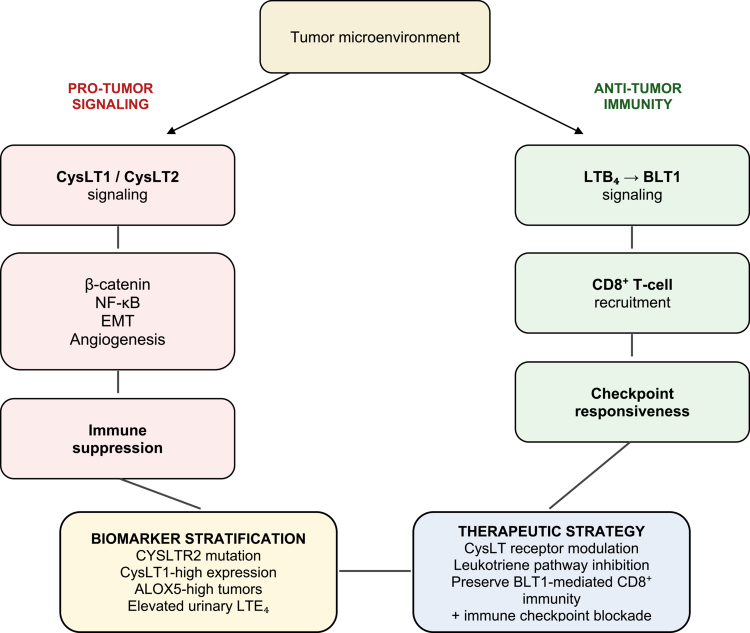
Biomarker-guided leukotriene targeting in the tumor microenvironment. Elevated leukotriene signaling contributes to tumor progression through CysLT1/CysLT2-mediated activation of *β*-catenin, NF-κB, epithelial–mesenchymal transition, angiogenesis, and immune suppression. Conversely, LTB₄–BLT1 signaling promotes CD8⁺ T-cell recruitment and supports responsiveness to immune checkpoint inhibition. Biomarker-based stratification, including CYSLTR2 mutations, CysLT1 overexpression, ALOX5-high tumors, and elevated urinary LTE₄, may enable receptor-selective therapeutic approaches while preserving BLT1-mediated antitumor immunity. Combination strategies with immune checkpoint blockade represent a potential precision-oncology framework for leukotriene pathway targeting.

In practical terms, biomarker-guided stratification could proceed as follows: (i) CYSLTR2 L129Q-mutant uveal melanoma → CYSLTR2 modulation (with or without CysLT1R antagonism), as supported by the translational case report above; (ii) CysLT1R-high tumors (e.g., colorectal, prostate, breast, lung) → CysLT1 antagonism; (iii) ALOX5-high or myeloid-inflamed tumors → 5-LO/FLAP inhibition combined with immune checkpoint blockade, while preserving LTB₄–BLT1 immunity; and (iv) urinary LTE₄ as a noninvasive pharmacodynamic biomarker for patient selection and target engagement. This stratification logic is summarized in Figure 2.

## Conclusion and future directions

Our analysis highlights leukotrienes as influential regulators of both tumor biology and immune activity. Acting through multiple receptor pathways, they regulate inflammation, proliferation, angiogenesis, and T-cell migration within the tumor microenvironment. CysLTs are typically pro-tumor, boosting inflammatory signals and inhibiting antitumor immunity, whereas LTB₄ enhances immune defense by guiding CD8⁺ T cells to tumor sites. These opposing functions underscore the complexity of inflammatory signaling in cancer, and leukotriene-directed therapies must consider both pro- and antitumor functions. The evidence supports a shift away from non-selective leukotriene inhibition toward receptor-specific and biomarker-guided strategies that limit tumor-promoting inflammation while preserving immunity.

Several caveats should frame this outlook. First, the great majority of mechanistic evidence is preclinical; current human data are predominantly pharmacoepidemiologic (e.g., reduced cancer incidence among leukotriene-modifier users) rather than from randomized trials, so mechanistic findings should not be read as established clinical efficacy. Second, the safety profile of available agents—notably the montelukast neuropsychiatric boxed warning and zileuton hepatotoxicity—must inform any oncologic repurposing.

We therefore propose specific, testable next steps. (1) Biomarker-stratified window-of-opportunity and phase II trials combining a CysLT1 antagonist with anti-PD-1 therapy in CysLT1R-high or ALOX5-high non-small-cell lung cancer, with on-treatment biopsies to confirm preserved CD8⁺ infiltration. (2) A CYSLTR2-mutant basket trial in uveal melanoma testing receptor-selective modulation. (3) Correlative incorporation of tumor ALOX5 expression and urinary LTE₄ as predictive and pharmacodynamic endpoints. (4) Formulation and dose-finding studies to determine whether tumor-relevant exposures can be achieved safely. (5) Explicit avoidance of non-selective LTB₄ blockade, which may impair antitumor immunity. Continued research into leukotriene biology, pursued through this receptor-resolved and biomarker-guided lens, will be critical for translating these insights into precision cancer therapies.

## Data Availability

No new datasets were created in this review. All relevant data are included in the references.
